# Seizure and Hepatosplenomegaly—Rare Manifestation of Parvovirus B-19: A Case Report and Review of the Literature

**DOI:** 10.1155/2011/287914

**Published:** 2011-06-01

**Authors:** Yadav Kamlesh, Gupta Pallav, Murari Manjula, Malik Rohan

**Affiliations:** ^1^Department of Pathology, Sanjay Gandhi Postgraduate Institute of Medical Sciences, Lucknow 226014, India; ^2^Department of Pediatric Gastroentology, Sanjay Gandhi Postgraduate Institute of Medical Sciences, Lucknow 226014, India

## Abstract

Parvovirus B19 is the etiologic agent of erythema infectiosum (fifth disease), a fever-rash illness occurring in childhood. We present a 10 month old child with high grade fever for 10 days, generalized tonic-clonic seizure, bilateral cervical lymphadenopathy, generalized maculopapular rash, hematemesis and malena. Bone marrow aspiration and liver biopsy were done. EBV serology and parvovirus PCR were also performed. Bone marrow aspiration and biopsy showed giant pro-erythroblast consistent with parvovirus infection. PCR showed amplification of parvovirus genomic sequences. Present case highlights an atypical presentation of Parvovirus B19 infection as fever, rash and hepatosplenomegaly.

## 1. Introduction

Parvovirus B19 is the etiologic agent of erythema infectiosum (fifth disease), a fever/rash illness occurring in childhood. In adults it causes varying degree of aplastic anemia usually in immunocompromised hosts due to organ transplant or immunodeficiency syndromes. Systemic manifestation of infection includes multisystem involvement and viral hemophagocytic syndrome.

Hepatitis and encephalitis resulting in hepatosplenemegaly and seizure may also be caused by parvovirus, however incidence is very rare [1]. Very few case reports exist in the literature with a clinical manifestation of fever, rash, hepatosplenomegaly, and seizure as a result of parvovirus infection.

Herein we describe a case presenting with fever, rash, hepatosplenomegaly, and seizure due to parvovirus infection.

## 2. Case Report

A 10-month-old child presented with high-grade fever for 10 days followed by three episodes of generalized tonic-clonic seizure. Subsequently patient developed bilateral cervical lymphadenopathy, generalized maculopapular rash, hematemesis, and melena. On examination child was irritable with weight 7.5 kg, height 71 cm, head circumference 41 cm, mild pallor, and healed ulcers in oral cavity. Liver span was 4.5 cm firm with rounded margin, coarse ecotecture on USG, and spleen was palpable 4.0 cm below left costal margin.

**Figure 1:**
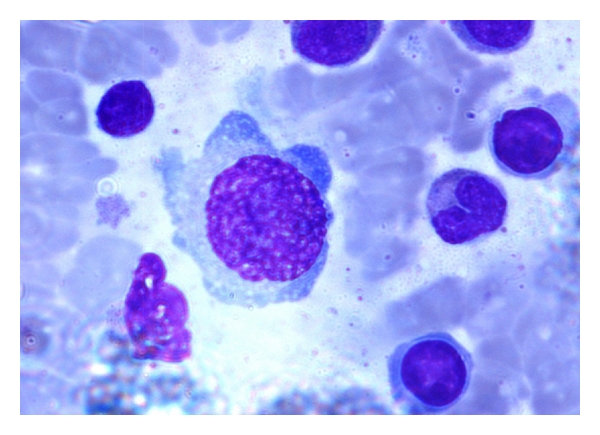
Bone marrow aspiration showing giant proerythroblast with prominent nucleoli and cytoplasmic blebs. Myeloid cells were relatively increased.

**Figure 2:**
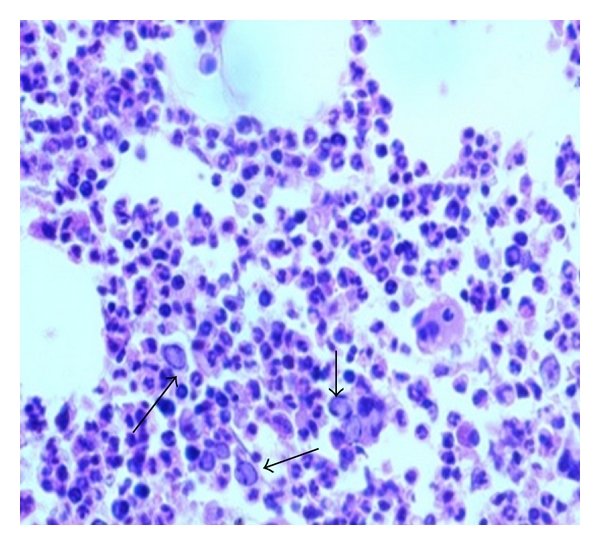
Bone marrow biopsy showing giant proerythroblast, nuclear clearing with eosinophilic intranuclear inclusion and peripheral condensation of chromatin. Late erythroid precursors are reduced, and myeloid cells are increased.

With the previous presentation, differential diagnosis of postviral hemophagocytosis/infiltrative disorders was kept. On investigation total leukocyte count was high with lymphocytosis, and peripheral smear showed activated lymphocytes. Fibrinogen was low, ferritin was high, and triglyceride was raised (Table 1). Bone marrow aspiration and liver biopsy were done. EBV serology and parvovirus PCR were also performed. Bone marrow aspiration and biopsy showed giant proerythroblast consistent with parvovirus infection (Figures 1 and 2). CSF culture was sterile. PCR showed amplification of parvovirus genome. Upper gastrointestinal endoscopy was done and was normal. No abnormality was detected to explain hematemesis. Liver biopsy showed mild nonspecific steatohepatitis. Serology for human immunodeficiency virus was negative. 

Patient was managed conservatively with oral antibiotic, vitamins, and hematinics. Child was afebrile at discharge with persistent hepatosplenomegaly.

At follow up patient was afebrile. On ultrasonography liver span and spleen were normal. The hematological and biochemical parameters were within normal limit.

## 3. Discussion

Acute infection of parvovirus in childhood causes fever rash illness known as “erythema infectiusum” or “fifth disease”. Disease is characterized by fever, rash, constitutional symptoms and is self-limiting. In immunocompetent adult hosts virus causes acute symmetric polyarthropathy. In immunocompromised adult host chronic infection of parvovirus B19 causes variable degree of erythroid hypoplasia and in severe form leads to pure red cell aplasia. The pathogenesis may be due to its tropism and direct cytotoxicity to erythroid progenitor cells [2].

Nervous system involvement of parvovirus infection is rare and usually manifest as meningitis, encephalitis, and very rarely seizure episode [3, 4]. Hepatic involvement is also very rare and usually seen in cases of coinfection with other hepatotropic viruses [1]. Simultaneous involvement of CNS and liver resulting in encephalitis, hepatitis, and hepatosplenomegaly is exceptionally rare, and very few case reports are available in the literature [1]. Parvovirus causes anemia due to selective erythroid tropism resulting in hypoplasia. Exact pathologic mechanism of erythroid hypoplasia is not clear; however receptor for virus on erythroid precursor cells is a likely mechanism. Pathologic mechanism of meningitis, encephalitis, and hepatitis is also unclear. This immunocompetent child presented with self limiting fever, hepatitis, hepatosplenomegaly, and seizure. He was managed conservatively and was stable at discharge.

## 4. Conclusion

We present this case of 10-month-old immunocompetent male child presenting with seizure, fever, rash with a bone marrow and PCR diagnosis of parvovirus infection for its extremely rare presentation with encephalitis and hepatosplenomegaly. 

In the light of the present experience, the diagnosis of parvovirus B19 acute infection should be considered in any case of acute febrile illness with seizure, rash, and hepatosplenomegaly.

## Figures and Tables

**Table 1:** Laboratory profile.

Hemogram	Urine	Blood chemistry
Hb = 9.7 gm/dl, TLC =	Culture: positive for	Total protein = 6.4 g/dL
37000/cumm	Klebsiella pneumonae.	Albumin = 2.6 g/dL
PLT : 2,44,000/cumm	BACTEC (aerobic)	Amylase = 44 U/L
DLC : P24, L71, E01,	bacterial culture	SGOT = 131 U/L
M02,	sensitivity:	SGPT = 105 U/L
Red cell indices	Coagulase negative	GGT = 802 U/L
(1) MCV = 79 fl,	Staphylococcus, sensitive	LDH = 695 U/L
(2) MCH = 25.6 pg,	to Vancomycin.	Alkaline phosphatase: 469
(3) MCHC = 32.4%,		Total bilirubin: 1.2 mg/dL
Reti = 0.5		Conjugated bilirubin =
PS: normocytic		0.7 mg/dL
hypochromic		Fibrinogen: 122 mg/dL
		Ferritin: 822 *μ*g/L

**Table 2:** Laboratory profile.

Serology	Bone marrow (BM)	Liver biopsy
EBV ELISA: negative	BM aspiration and biopsy: giant	Nonspecific steatohepatitis
HAV, HBV, HCV:	proerythroblasts, s/o parvovirus	
negative	infection	
Parvovirus PCR: positive		
